# Association of preoperative systemic immune-inflammation index and geriatric nutritional risk index with prolonged postoperative length of stay in older patients with hip fracture: A retrospective cohort study

**DOI:** 10.1097/MD.0000000000051293

**Published:** 2026-10-23

**Authors:** Jinguang Zhang, Xiao Yu, Dongxu Sun

**Affiliations:** a Department of Anesthesiology, Hebei Cangzhou Hospital of Integrated Traditional Chinese and Western Medicine, Cangzhou, Hebei, China; b Department of Neurology Intervention, Cangzhou People’s Hospital, Cangzhou, Hebei, China.

**Keywords:** geriatric nutritional risk index, hip fracture, interaction, prolonged postoperative length of stay, systemic immune-inflammation index

## Abstract

This study aimed to evaluate the associations of the preoperative systemic immune-inflammation index (SII), geriatric nutritional risk index (GNRI), and their joint exposure with prolonged postoperative length of stay (LOS) in older patients undergoing hip fracture surgery. This retrospective cohort study included older patients who underwent surgery for hip fracture. SII was calculated from preoperative platelet, neutrophil, and lymphocyte counts, and GNRI from albumin and body weight. High SII was defined as above the cohort-specific 75th percentile, GNRI ≤98 as nutritional risk, and prolonged postoperative LOS as above the cohort-specific 75th percentile. Logistic regression was used to assess associations of SII, GNRI, and their joint exposure with prolonged postoperative LOS, adjusting for age, sex, Charlson comorbidity index, fracture type, and preoperative waiting time. Interaction, discrimination, subgroup, and sensitivity analyses were also performed. Among 653 patients, higher log(SII) was associated with greater odds of prolonged postoperative LOS (OR = 2.23, 95% confidence interval [CI] 1.68–2.94), as was each 10-point decrease in GNRI (OR = 1.84, 95% CI 1.26–2.68). Compared with patients with non-high SII and no nutritional risk, those with both high SII and GNRI-defined nutritional risk had substantially higher odds of prolonged postoperative LOS (OR = 10.16, 95% CI 5.52–18.67). Each 1-point increase in the SII-GNRI risk burden score was associated with higher odds (OR = 3.30, 95% CI 2.42–4.51; *P* for trend <.001). Multiplicative interaction was not significant (*P* = .138), whereas exploratory OR-based additive interaction measures were positive. The clinical model area under the receiver operating characteristic curve increased from 0.697 to 0.774 after adding SII-GNRI joint exposure. Findings were generally consistent across subgroup and sensitivity analyses. Coexisting high SII and GNRI-defined nutritional risk may identify older patients with higher odds of prolonged postoperative LOS after hip fracture surgery. Joint assessment of inflammatory and nutritional status may provide additional information for perioperative risk stratification. Given the single-center retrospective design, cohort-derived thresholds, and lack of validation, these findings are preliminary and require confirmation in independent populations.

## 1. Introduction

Hip fracture is one of the most severe fragility fractures in older adults and is associated with increased mortality, impaired functional recovery, loss of independence, and substantial healthcare burden. Feng et al analyzed the Global Burden of Disease 2019 data across 204 countries and territories and showed that the global burden of hip fracture in adults aged 55 years and older remains considerable.^[1]^ Dyer et al reviewed 38 studies and reported persistent impairment in mobility, activities of daily living, health-related quality of life, and independent living after hip fracture.^[2]^ Moja et al found in a meta-analysis of more than 190,000 patients that surgery within 48 hours was associated with better outcomes.^[3]^ A subsequent international randomized trial further evaluated the clinical effect of accelerated surgery in this population.^[4]^ These findings indicate that postoperative recovery is influenced not only by surgical timing and care pathways, but also by the intrinsic biological reserve of the patient before surgery.

Inflammatory immune burden and nutritional risk are 2 clinically relevant dimensions of this biological reserve. Furman et al summarized the role of chronic inflammation in age-related disease and functional decline.^[5]^ Ferrucci and Fabbri further described inflammageing as a biological background for frailty, cardiovascular disease, and mortality.^[6]^ The systemic immune-inflammation index (SII) is derived from platelet, neutrophil, and lymphocyte counts (LYM) and reflects thrombo-inflammatory activation, innate immune response, and impaired immune regulation. Hu et al first proposed SII and demonstrated its prognostic value in hepatocellular carcinoma.^[7]^ In older patients with hip fracture, Wang et al reported that SII independently predicted postoperative survival.^[8]^ Nutritional risk is also closely linked to hip fracture prognosis. Bouillanne et al developed the geriatric nutritional risk index (GNRI) for nutritional risk assessment in hospitalized older adults.^[9]^ Avenell et al showed in a Cochrane review that inadequate nutritional intake is common after hip fracture and that supplementation may affect recovery and complications.^[10]^ Funahashi et al reported that GNRI predicted 30-day mortality after hip fracture surgery.^[11]^ Chiavarini et al also showed that malnutrition worsens mortality, mobility, and independence in older adults with hip fracture.^[12]^

Although previous studies have examined SII and GNRI separately, inflammatory immune burden and nutritional risk are not independent clinical states. Inflammation can intensify catabolism and impair tissue repair, while nutritional risk may weaken immune regulation and postoperative recovery capacity. Zhou et al combined SII and GNRI to predict 1-year survival after surgery for hip fracture, suggesting the potential value of a combined inflammatory-nutritional assessment.^[13]^ However, most existing studies have focused on mortality, delirium, or complications. Evidence remains limited regarding prolonged postoperative length of stay (LOS), an outcome that more directly reflects early perioperative recovery and discharge readiness. Whether high SII and GNRI-defined nutritional risk exert a synergistic effect on prolonged postoperative LOS also remains unclear. Therefore, this study evaluated the associations of preoperative SII, GNRI, and SII-GNRI joint exposure with prolonged postoperative LOS in older patients with hip fracture, and further assessed the multiplicative and additive interactions between high SII and GNRI-defined nutritional risk.

## 2. Materials and methods

### 2.1. Study design and participants

This retrospective cohort study consecutively included older patients who underwent surgical treatment for hip fracture at Hebei Cangzhou Hospital of Integrated Traditional Chinese and Western Medicine between February 2025 and December 2025. The inclusion criteria were age ≥65 years, radiographic diagnosis of femoral neck fracture, intertrochanteric fracture, or subtrochanteric fracture, and receipt of surgical treatment. The exclusion criteria were pathological fracture, old fracture, multiple severe trauma, conservative treatment, missing key preoperative laboratory variables, and incomplete outcome data. The participant selection process and reasons for exclusion are shown in Figure 1. The study size was determined by including all consecutive eligible patients who met the predefined inclusion and exclusion criteria during the study period. Because this was a retrospective observational cohort study, no formal sample size calculation was performed before study initiation.

**Figure 1.**
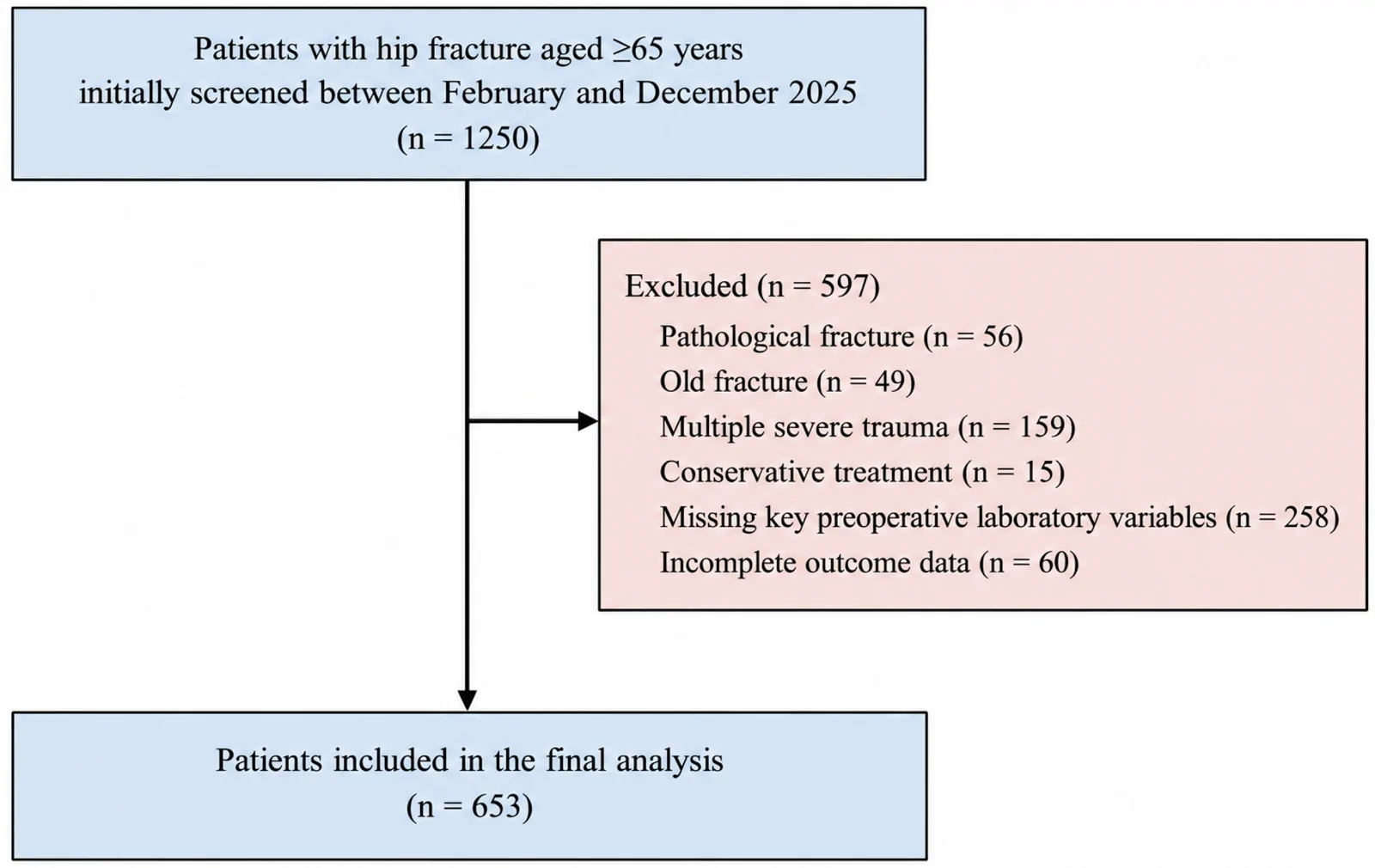
Participant flow diagram.

The study was approved by the Ethics Committee of Hebei Cangzhou Hospital of Integrated Traditional Chinese and Western Medicine (approval No. CZX2024-KY-089.1) and was conducted in accordance with the Declaration of Helsinki. Given the retrospective design and the use of de-identified patient data, the requirement for informed consent was waived by the Ethics Committee.

### 2.2. Data collection

Demographic data, comorbidities, perioperative characteristics, laboratory variables, and hospitalization outcomes were obtained from the electronic medical record system. Demographic variables included age, sex, and body mass index. Comorbidities included hypertension, diabetes mellitus, coronary heart disease, cerebrovascular disease, chronic kidney disease, chronic obstructive pulmonary disease, and cognitive impairment or dementia. The Charlson comorbidity index (CCI) was calculated. Perioperative variables included American Society of Anesthesiologists (ASA) classification, fracture type, surgical procedure, anesthesia type, preoperative waiting time, operative duration, intraoperative blood loss, and perioperative transfusion. Preoperative waiting time was defined as the interval from hospital admission to surgery and was calculated as total hospital LOS minus postoperative LOS. Preoperative laboratory variables included albumin (Alb), platelet count (PLT), neutrophil count (NEUT), LYM, hemoglobin (Hb), estimated glomerular filtration rate (eGFR), glucose (Glu), and D-dimer. Of the 1250 patients initially screened, 258 (20.6%) were excluded because one or more key preoperative laboratory variables were unavailable, and 60 (4.8%) were excluded because outcome data were incomplete. No imputation was performed, and the analysis was based on complete cases. Because key laboratory or outcome information was unavailable for these excluded patients, a comprehensive comparison with the included cohort was not feasible.

### 2.3. Definitions of SII, GNRI, and joint exposure

SII was calculated using preoperative PLT, NEUT, and LYM according to the formula SII = PLT × NEUT/LYM. Because SII had a right-skewed distribution, log-transformed SII was used in the continuous-variable analysis. For categorical analyses, high SII was defined as an SII value above the cohort-specific 75th percentile (1102.5).

GNRI was used to evaluate nutritional risk and was calculated as GNRI = 1.489 × Alb (g/L) + 41.7 × minutes (1, actual body weight/ ideal body weight). Ideal body weight was calculated using the Lorentz formula. GNRI ≤ 98 was defined as nutritional risk according to the original GNRI risk classification.^[9]^

Patients were classified into 4 SII-GNRI joint exposure groups according to high SII status and GNRI-defined nutritional risk: non-high SII plus no nutritional risk, high SII plus no nutritional risk, non-high SII plus GNRI-defined nutritional risk, and high SII plus GNRI-defined nutritional risk. The SII-GNRI risk burden score was further constructed as the sum of 2 binary indicators (high SII and GNRI-defined nutritional risk), with possible values of 0, 1, and 2 points, and was used for trend testing.

### 2.4. Outcome definition

The primary outcome was prolonged postoperative LOS. Postoperative LOS was defined as the number of days from surgery to hospital discharge. The 75th percentile of postoperative LOS in the study cohort was 12 days, and prolonged postoperative LOS was defined as LOS > 12 days. In the sensitivity analysis, prolonged postoperative LOS was alternatively defined as postoperative LOS > 15 days, corresponding to the 90th percentile of the cohort. No in-hospital deaths, transfers to other hospitals, or discharges against medical advice occurred in the analytic cohort.

### 2.5. Statistical analysis

Continuous variables were summarized as mean ± standard deviation or median with interquartile range according to distributional characteristics. Categorical variables were summarized as number and percentage. Baseline characteristics across the 4 joint exposure groups were compared using the Kruskal-Wallis test for continuous variables and Pearson’s chi-square test with Monte Carlo simulation for categorical variables, as appropriate.

Logistic regression models were used to examine the associations of SII, GNRI, and SII-GNRI joint exposure with prolonged postoperative LOS. Model 1 was unadjusted. Model 2 was adjusted for age, sex, CCI, fracture type, and preoperative waiting time. Covariates were selected a priori according to their temporal relationships with the exposures and outcome and their plausible roles as common causes of preoperative inflammatory-nutritional status and postoperative LOS. A conceptual causal framework was used to illustrate the rationale underlying covariate selection (Fig. 2). Variables that were components of SII or GNRI, as well as physiological or treatment-related factors that could lie on downstream pathways after exposure assessment, were not included in the primary adjustment set to minimize potential overadjustment. Results were reported as odds ratios (ORs) with 95% confidence intervals (CIs). In continuous-variable analyses, log(SII) and each 10-point decrease in GNRI were assessed separately. In joint exposure analyses, the non-high SII plus no nutritional risk group was used as the reference. Trend testing was based on the SII-GNRI risk burden score modeled as a continuous-variable. Multiplicative interaction between high SII and GNRI-defined nutritional risk was assessed by adding the high SII×GNRI-defined nutritional risk interaction term to the logistic regression model. Additive interaction was explored using the relative excess risk due to interaction (RERI), attributable proportion due to interaction (AP), and synergy index (SI), derived from logistic regression ORs. Because prolonged postoperative LOS was not a rare outcome, these OR-based additive interaction measures were considered approximate and were interpreted descriptively and exploratory rather than as direct estimates of risk-based additive interaction.

**Figure 2.**
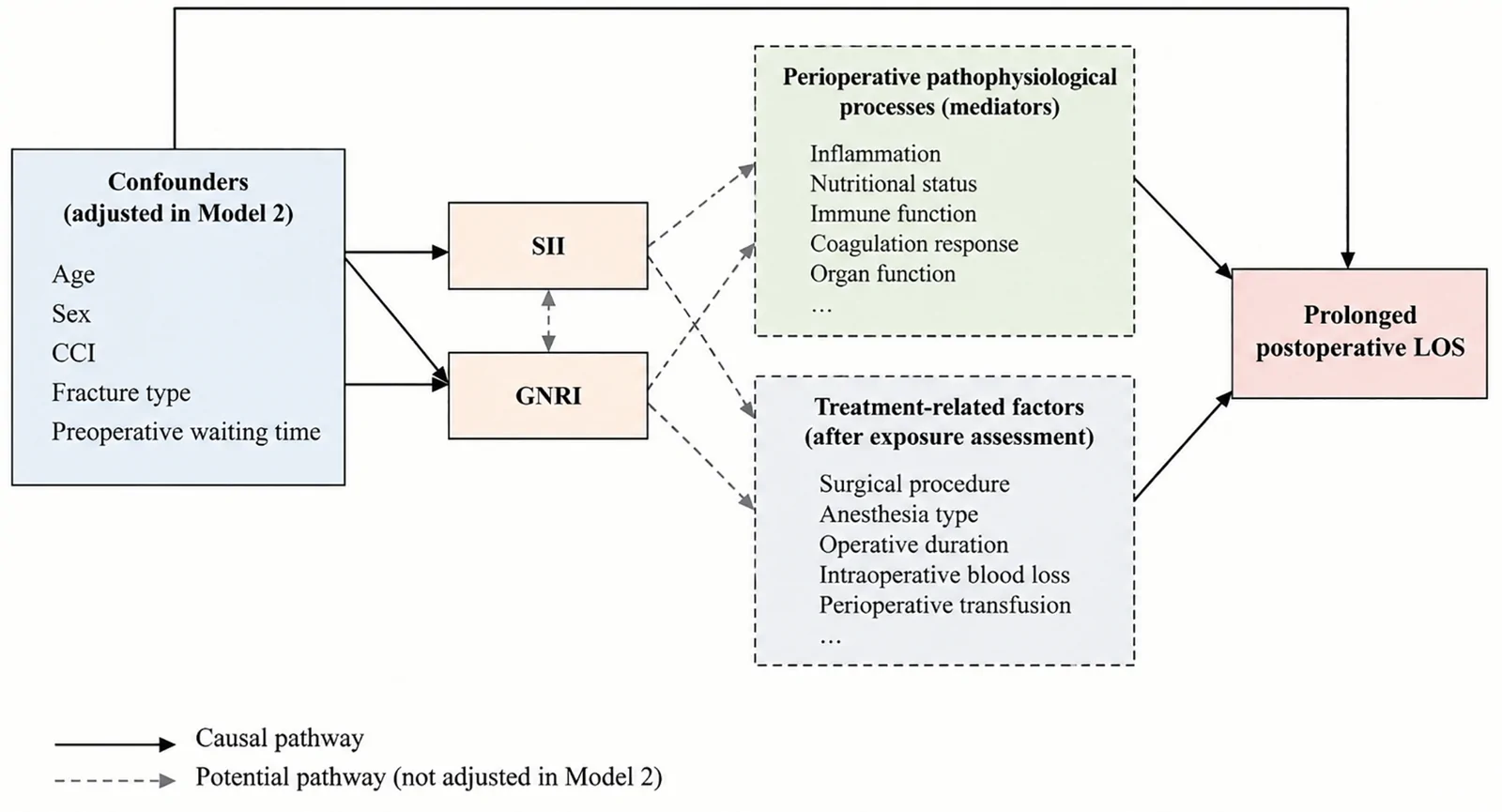
Conceptual causal framework illustrating the rationale for covariate selection. Solid arrows represent hypothesized causal relationships. Age, sex, CCI, fracture type, and preoperative waiting time were included in the primary adjustment set. Variables considered potential intermediates or treatment-related factors were not included in Model 2. CCI = Charlson comorbidity index, GNRI = geriatric nutritional risk index, LOS = length of stay, SII = systemic immune-inflammation index.

To evaluate incremental discrimination, receiver operating characteristic curves were plotted and areas under the curve (AUCs) with 95% CIs were calculated. The clinical model included the covariates used in Model 2. SII, GNRI, SII plus GNRI, and SII-GNRI joint exposure were then added to the clinical model. AUCs were compared using the DeLong test. Subgroup analyses were performed by age, sex, ASA classification, fracture type, and anesthesia type. These subgroup analyses were considered exploratory, and no adjustment for multiple comparisons was applied. Subgroup models were adjusted using the same covariate set as Model 2; when the stratification variable was itself included in the Model 2 adjustment set, it was omitted from the corresponding subgroup model. Interaction *P* values were obtained using likelihood ratio tests comparing models with and without the interaction term. Sensitivity analyses were performed by redefining high SII using the cohort-specific median and by redefining prolonged postoperative LOS as postoperative LOS above the 90th percentile. Multicollinearity was assessed using the variance inflation factor (VIF) or adjusted generalized VIF, as appropriate. Statistical analyses were conducted using R version 4.4.3. All tests were two-sided, and *P* < .05 was considered statistically significant.

## 3. Results

### 3.1. Baseline characteristics

A total of 653 older patients who underwent surgery for hip fracture were included. The median postoperative LOS was 10 days (interquartile range, 8–12 days). The 75th percentile of postoperative LOS was 12 days, which was used as the cutoff for defining prolonged postoperative LOS. The 90th percentile cutoff was 15 days and was applied in the sensitivity analysis. The 75th percentile of preoperative SII was 1102.5, and 163 patients were classified as having high SII. GNRI-defined nutritional risk was present in 366 patients. According to SII and GNRI status, 249 patients (38.1%) were classified as non-high SII plus no nutritional risk, 38 (5.8%) as high SII plus no nutritional risk, 241 (36.9%) as non-high SII plus GNRI-defined nutritional risk, and 125 (19.1%) as high SII plus GNRI-defined nutritional risk.

Baseline characteristics are shown in Table 1. The 4 groups differed significantly in age, sex, body mass index, SII, GNRI, Alb, PLT, NEUT, LYM, Hb, eGFR, Glu, D-dimer, ASA classification, CCI, preoperative waiting time, and several comorbidities. Overall, patients with both high SII and GNRI-defined nutritional risk were older, had lower GNRI, Alb, Hb, and eGFR, had higher SII, PLT, NEUT, Glu, and D-dimer, and had higher ASA classification and CCI. Fracture type, surgical procedure, anesthesia type, operative duration, intraoperative blood loss, and perioperative transfusion did not differ significantly across groups.

**Table 1:** Baseline characteristics according to SII-GNRI joint exposure groups.

Variable	Overall N = 653	G1 N = 249	G2 N = 38	G3 N = 241	G4 N = 125	*P* value
Age, yr	81.8 ± 7.3	78.3 ± 6.7	81.2 ± 5.6	83.1 ± 7.0	86.6 ± 6.1	<.001
Sex						.008
Female	442 (67.7%)	175 (70.3%)	32 (84.2%)	146 (60.6%)	89 (71.2%)	
Male	211 (32.3%)	74 (29.7%)	6 (15.8%)	95 (39.4%)	36 (28.8%)	
Body mass index, kg/m^2^	22.8 ± 2.5	23.7 ± 2.1	22.9 ± 2.0	22.4 ± 2.4	21.9 ± 3.1	<.001
Systemic immune-inflammation index	662.0 (373.4, 1102.5)	413.0 (267.6, 595.6)	1468.6 (1173.3, 1816.6)	609.8 (392.7, 834.2)	1690.1 (1338.0, 2530.0)	<.001
Geriatric nutritional risk index	96.8 ± 5.9	102.1 ± 2.9	100.8 ± 2.5	93.5 ± 3.8	91.5 ± 4.8	<.001
Albumin, g/L	37.9 ± 3.3	40.9 ± 2.0	40.2 ± 1.7	35.9 ± 2.1	35.2 ± 2.4	<.001
Platelet count, 10^9^/L	232.8 ± 57.2	210.5 ± 52.0	276.9 ± 45.8	221.6 ± 48.3	285.2 ± 45.4	<.001
Neutrophil count, 10^9^/L	4.2 ± 1.3	3.6 ± 1.1	5.4 ± 0.8	4.0 ± 1.0	5.5 ± 0.9	<.001
Lymphocyte count, 10^9^/L	1.5 ± 0.5	1.8 ± 0.4	1.0 ± 0.3	1.6 ± 0.4	0.9 ± 0.3	<.001
Hemoglobin, g/L	123.4 ± 13.1	128.1 ± 11.3	121.0 ± 10.6	122.2 ± 13.2	117.2 ± 13.5	<.001
Estimated glomerular filtration rate, mL/min/1.73 m^2^	65.9 ± 17.0	73.1 ± 14.7	68.6 ± 15.4	63.4 ± 15.8	55.5 ± 17.3	<.001
Glucose, mmol/L	6.1 ± 1.1	5.9 ± 1.0	6.4 ± 1.1	6.1 ± 1.1	6.6 ± 1.1	<.001
D-dimer, mg/L FEU	2.0 (1.4, 2.9)	1.7 (1.1, 2.4)	3.0 (1.6, 3.9)	1.9 (1.4, 2.8)	2.6 (1.8, 3.6)	<.001
ASA classification						<.001
I-II	457 (70.0%)	205 (82.3%)	30 (78.9%)	168 (69.7%)	54 (43.2%)	
III-IV	196 (30.0%)	44 (17.7%)	8 (21.1%)	73 (30.3%)	71 (56.8%)	
Charlson Comorbidity Index	1.0 (1.0, 3.0)	1.0 (0.0, 2.0)	1.0 (1.0, 2.0)	2.0 (1.0, 3.0)	3.0 (1.0, 4.0)	<.001
Hypertension	334 (51.1%)	103 (41.4%)	24 (63.2%)	119 (49.4%)	88 (70.4%)	<.001
Diabetes mellitus	179 (27.4%)	54 (21.7%)	15 (39.5%)	61 (25.3%)	49 (39.2%)	.001
Coronary heart disease	204 (31.2%)	61 (24.5%)	7 (18.4%)	79 (32.8%)	57 (45.6%)	<.001
Cerebrovascular disease	174 (26.6%)	45 (18.1%)	13 (34.2%)	62 (25.7%)	54 (43.2%)	<.001
Chronic kidney disease	126 (19.3%)	26 (10.4%)	5 (13.2%)	56 (23.2%)	39 (31.2%)	<.001
Chronic obstructive pulmonary disease	97 (14.9%)	26 (10.4%)	4 (10.5%)	43 (17.8%)	24 (19.2%)	.045
Dementia	116 (17.8%)	29 (11.6%)	1 (2.6%)	48 (19.9%)	38 (30.4%)	<.001
Fracture type						.964
Femoral neck fracture	264 (40.4%)	101 (40.6%)	17 (44.7%)	97 (40.2%)	49 (39.2%)	
Intertrochanteric fracture	348 (53.3%)	131 (52.6%)	20 (52.6%)	128 (53.1%)	69 (55.2%)	
Subtrochanteric fracture	41 (6.3%)	17 (6.8%)	1 (2.6%)	16 (6.6%)	7 (5.6%)	
Surgical procedure						.707
Hemiarthroplasty	199 (30.5%)	86 (34.5%)	14 (36.8%)	64 (26.6%)	35 (28.0%)	
Internal fixation	370 (56.7%)	133 (53.4%)	19 (50.0%)	145 (60.2%)	73 (58.4%)	
Intramedullary nailing	37 (5.7%)	14 (5.6%)	1 (2.6%)	15 (6.2%)	7 (5.6%)	
Total hip arthroplasty	47 (7.2%)	16 (6.4%)	4 (10.5%)	17 (7.1%)	10 (8.0%)	
Anesthesia type						.537
General anesthesia	270 (41.3%)	98 (39.4%)	13 (34.2%)	102 (42.3%)	57 (45.6%)	
Regional anesthesia	383 (58.7%)	151 (60.6%)	25 (65.8%)	139 (57.7%)	68 (54.4%)	
Preoperative waiting time, days	2.0 (2.0, 3.0)	2.0 (2.0, 3.0)	2.0 (1.3, 3.0)	2.0 (2.0, 3.0)	2.0 (2.0, 3.0)	.020
Operative duration, min	85.0 (75.0, 100.0)	85.0 (75.0, 100.0)	85.0 (65.0, 95.0)	85.0 (75.0, 95.0)	80.0 (75.0, 100.0)	.439
Intraoperative blood loss, mL	200.0 (130.0, 280.0)	200.0 (130.0, 280.0)	210.0 (130.0, 300.0)	190.0 (130.0, 270.0)	200.0 (130.0, 280.0)	.667
Perioperative transfusion	12 (1.8%)	3 (1.2%)	0 (0.0%)	6 (2.5%)	3 (2.4%)	.585

G1, non-high SII plus no nutritional risk. G2, high SII plus no nutritional risk. G3, non-high SII plus GNRI-defined nutritional risk. G4, high SII plus GNRI-defined nutritional risk. Continuous variables are presented as mean ± standard deviation or median with interquartile range. Categorical variables are presented as number and percentage. Alb = albumin, ASA = American Society of Anesthesiologists.

BMI = body mass index, CCI = Charlson comorbidity index, COPD = chronic obstructive pulmonary disease, eGFR = estimated glomerular filtration rate, FEU = fibrinogen equivalent units, Glu = glucose, GNRI = geriatric nutritional risk index, Hb = hemoglobin, LYM = lymphocyte count, N = number of patients, NEUT = neutrophil count, PLT = platelet count, SII = systemic immune-inflammation index.

### 3.2. Associations of SII and GNRI with prolonged postoperative LOS

With prolonged postoperative LOS as the outcome, log(SII) and each 10-point decrease in GNRI were evaluated separately. Table 2 shows that higher log(SII) was associated with higher odds of prolonged postoperative LOS in Model 1 (OR = 2.67, 95% CI 2.06 to 3.46, *P* < .001), and the association remained after adjustment in Model 2 (OR = 2.23, 95% CI 1.68 to 2.94, *P* < .001). Each 10-point decrease in GNRI was also associated with higher odds of prolonged postoperative LOS, with ORs of 2.65 (95% CI 1.93 to 3.64, *P* < .001) in Model 1 and 1.84 (95% CI 1.26–2.68, *P* = .002) in Model 2. No evidence of problematic multicollinearity was observed in Model 2; VIF values ranged from 1.00 to 1.23, and the adjusted GVIF for fracture type was 1.003.

**Table 2:** Associations of SII and GNRI with prolonged postoperative LOS.

Indicator	Model 1 OR (95% CI)	*P* value	Model 2 OR (95% CI)	*P* value
log(SII)	2.67 (2.06, 3.46)	<.001	2.23 (1.68, 2.94)	<.001
Per 10-point decrease in GNRI	2.65 (1.93, 3.64)	<.001	1.84 (1.26, 2.68)	.002

Model 1 was unadjusted. Model 2 was adjusted for age, sex, CCI, fracture type, and preoperative waiting time.

CCI = Charlson comorbidity index, CI = confidence interval, GNRI = geriatric nutritional risk index, LOS = length of stay, OR = odds ratio, SII = systemic immune-inflammation index.

### 3.3. Joint exposure and interaction analyses

Using the non-high SII plus no nutritional risk group as the reference, the association between SII-GNRI joint exposure and prolonged postoperative LOS is shown in Table 3. In Model 2, high SII plus no nutritional risk was not significantly associated with prolonged postoperative LOS (OR = 1.99, 95% CI 0.78 to 5.09, *P* = .151). Non-high SII plus GNRI-defined nutritional risk was associated with higher odds of prolonged postoperative LOS (OR = 2.31, 95% CI 1.34 to 3.98, *P* = .003). The strongest association was observed in patients with both high SII and GNRI-defined nutritional risk (OR = 10.16, 95% CI 5.52 to 18.67, *P* < .001). The risk burden score showed a graded association with prolonged postoperative LOS. Each 1-point increase in the score was associated with higher odds after adjustment (OR = 3.30, 95% CI 2.42 to 4.51, *P* for trend <.001).

**Table 3:** Association of SII-GNRI joint exposure with prolonged postoperative LOS.

SII-GNRI joint exposure group	N	Prolonged postoperative LOS, n (%)	Model 2 OR (95% CI)	*P* value
Non-high SII+no nutritional risk	249	24 (9.6%)	Ref	
High SII+no nutritional risk	38	7 (18.4%)	1.99 (0.78, 5.09)	.151
Non-high SII+GNRI-defined nutritional risk	241	56 (23.2%)	2.31 (1.34, 3.98)	.003
High SII+GNRI-defined nutritional risk	125	74 (59.2%)	10.16 (5.52, 18.67)	<.001
SII-GNRI risk burden score, per 1-point increase	–	–	3.30 (2.42, 4.51)	<.001

Model 2 was adjusted for age, sex, CCI, fracture type, and preoperative waiting time. The SII-GNRI risk burden score was defined as the sum of 2 binary indicators (high SII and GNRI-defined nutritional risk).

CCI = Charlson Comorbidity Index, CI = confidence interval, GNRI = Geriatric Nutritional Risk Index, LOS = length of stay, N/n = number of patients, OR = odds ratio, Ref = reference group, SII = Systemic Immune-Inflammation Index.

Interaction analyses further evaluated the joint effect of high SII and GNRI-defined nutritional risk (Table 4). The multiplicative interaction was not statistically significant (OR = 2.21, 95% CI 0.77 to 6.31, *P* = .138). Additive interaction measures derived from logistic regression ORs showed positive estimates, with RERI of 6.86 (95% CI 1.66 to 12.05, *P* = .010), AP of 0.68 (95% CI 0.46–0.89, *P* < .001), and SI of 3.98 (95% CI 1.56 to 10.20, *P* = .004). However, because prolonged postoperative LOS was not a rare outcome, these OR-based additive interaction estimates should be interpreted cautiously as approximate measures rather than direct estimates of risk-based additive interaction, and the additive interaction findings should therefore be regarded as exploratory.

**Table 4:** Interaction between high SII and GNRI-defined nutritional risk

Interaction measure	Estimate (95% CI)	*P* value
Multiplicative interaction OR	2.21 (0.77, 6.31)	.138
RERI	6.86 (1.66, 12.05)	.010
AP	0.68 (0.46, 0.89)	<.001
SI	3.98 (1.56, 10.20)	.004

The model adjusted for age, sex, CCI, fracture type, and preoperative waiting time. Multiplicative interaction was assessed using the high SII×GNRI-defined nutritional risk interaction term. Additive interaction was assessed using RERI, AP, and SI. RERI, AP, and SI were interpreted descriptively only; because these measures were derived from logistic regression ORs and the outcome was not rare, the OR-based additive interaction estimates and associated *P* values were considered exploratory rather than confirmatory.

AP = attributable proportion due to interaction, CCI = Charlson Comorbidity Index, CI = confidence interval, GNRI = Geriatric Nutritional Risk Index, OR = odds ratio, RERI = relative excess risk due to interaction, SI = synergy index, SII = Systemic Immune-Inflammation Index.

### 3.4. Incremental discrimination of SII, GNRI, and joint exposure

receiver operating characteristic curve analysis is shown in Figure 3. The clinical model had an AUC of 0.697 (95% CI 0.650 to 0.743). After adding SII to the clinical model, the AUC increased to 0.748 (95% CI 0.704 to 0.792, DeLong *P* = .001). After adding GNRI, the AUC was 0.717 (95% CI 0.672 to 0.762, DeLong *P* = .034). The AUC increased to 0.752 (95% CI 0.708 to 0.796, DeLong *P* < .001) after adding both SII and GNRI. The model including SII-GNRI joint exposure showed the highest in-sample AUC of 0.774 (95% CI 0.731 to 0.817, DeLong *P* < .001).

**Figure 3.**
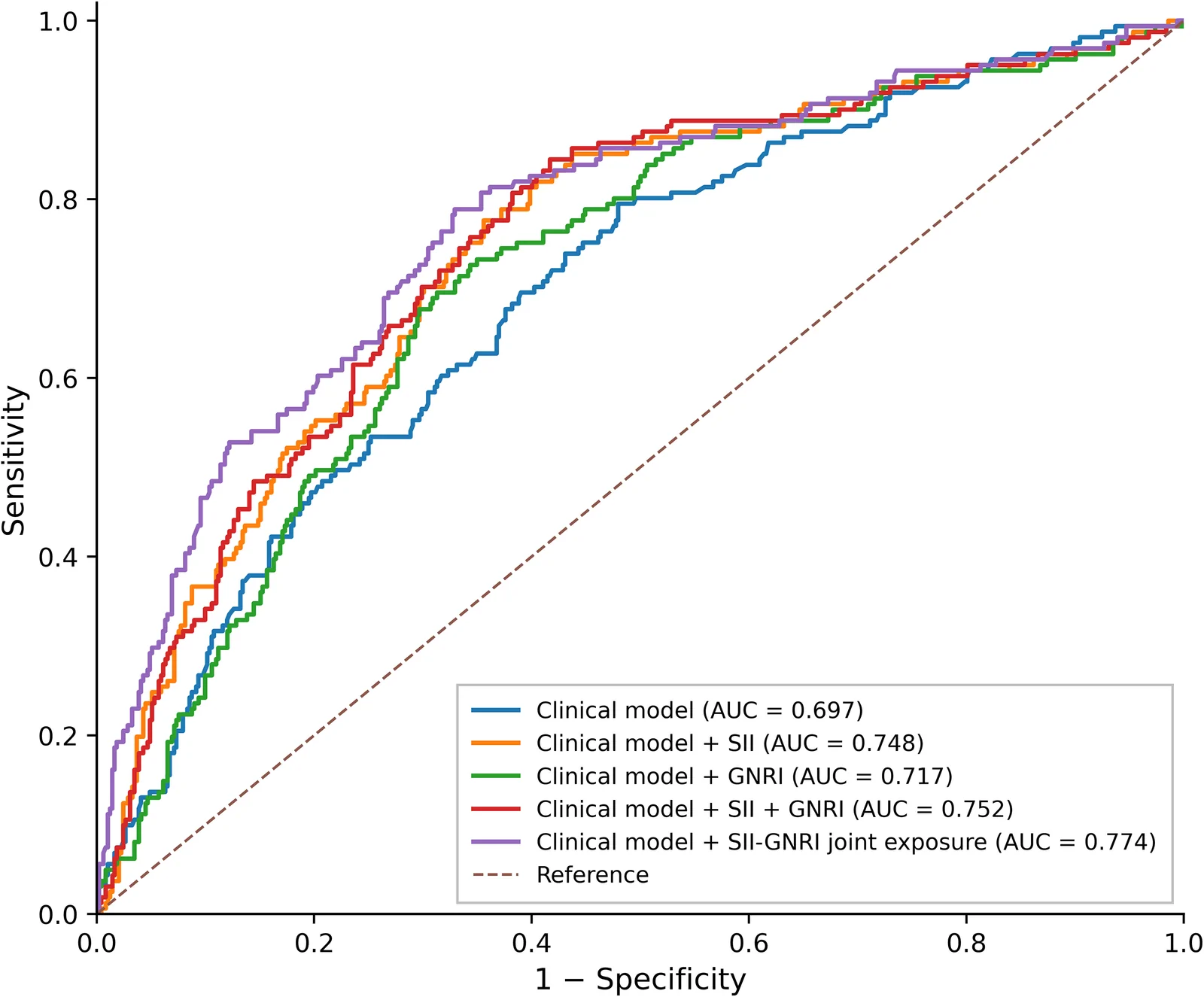
Receiver operating characteristic curves for models predicting prolonged postoperative LOS. he clinical model included age, sex, CCI, fracture type, and preoperative waiting time. AUC = area under the curve, CCI = Charlson comorbidity index, GNRI = geriatric nutritional risk index, LOS = length of stay, SII = systemic immune-inflammation index.

### 3.5. Subgroup analyses

Subgroup analyses are shown in Figure 4. Across strata of age, sex, ASA classification, fracture type, and anesthesia type, the associations of log(SII), each 10-point decrease in GNRI, and the joint high SII plus GNRI-defined nutritional risk indicator with prolonged postoperative LOS were generally directionally consistent. Although several subgroup-specific estimates did not reach statistical significance and some estimates were imprecise in smaller strata, none of the interaction tests was statistically significant (all *P* for interaction >.05). Thus, there was no statistical evidence of effect modification by age, sex, ASA classification, fracture type, or anesthesia type. These subgroup analyses were exploratory, and no adjustment for multiple comparisons was performed.

**Figure 4.**
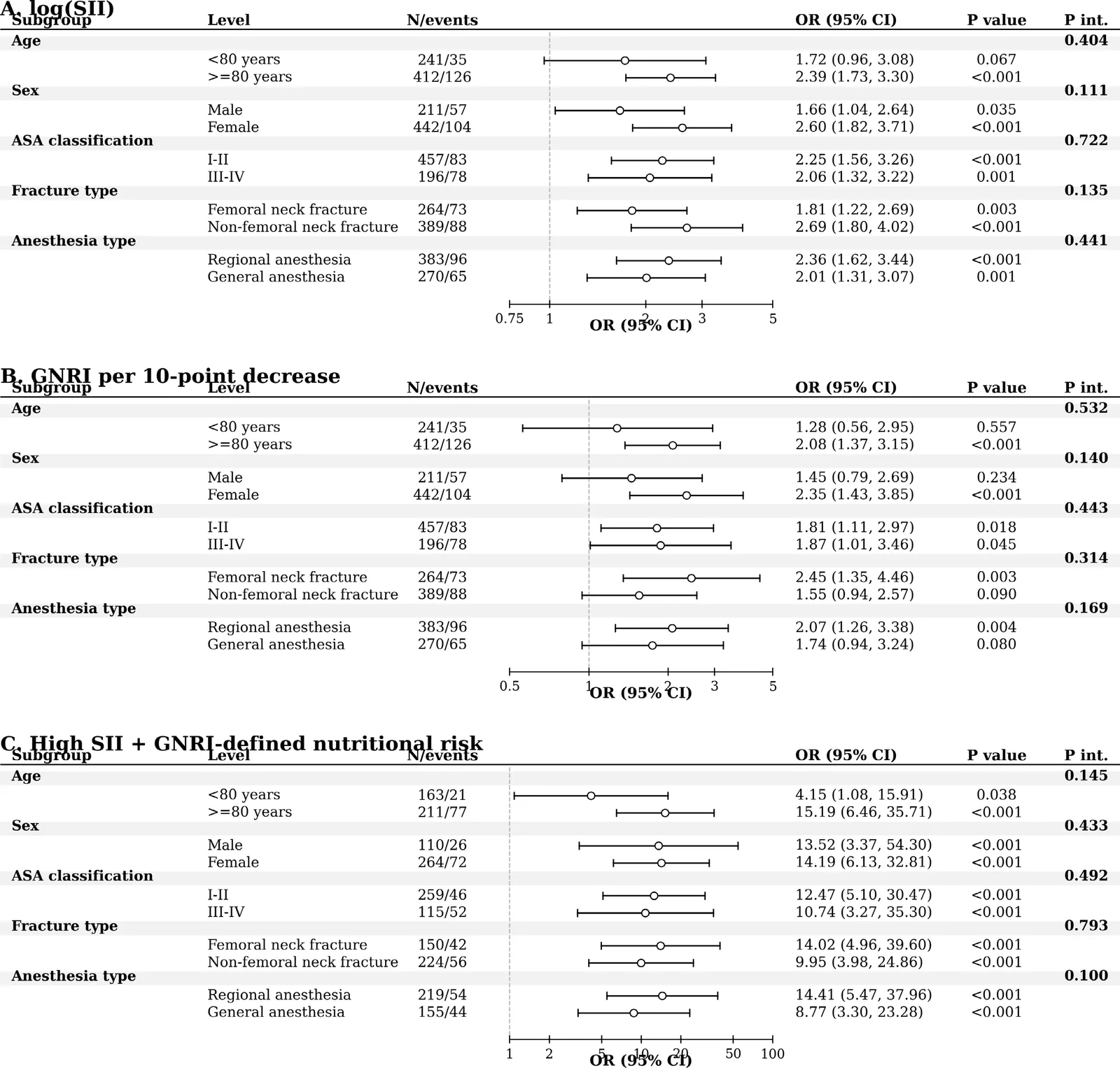
Subgroup analyses of inflammatory and nutritional indicators in relation to prolonged postoperative LOS. Models were adjusted for age, sex, CCI, fracture type, and preoperative waiting time. When age, sex, or fracture type was used as the stratification variable, the corresponding variable was omitted from the adjustment set. The joint indicator represents high SII plus GNRI-defined nutritional risk compared with non-high SII plus no nutritional risk. Subgroup analyses were exploratory, and no adjustment for multiple comparisons was performed. CCI = Charlson comorbidity index, CI = confidence interval, GNRI = geriatric nutritional risk index, LOS = length of stay, OR = odds ratio, P int = *P* value for interaction, SII = systemic immune-inflammation index.

### 3.6. Sensitivity analyses

Sensitivity analysis results are shown in Table 5. When high SII was redefined using the cohort-specific median, high SII plus GNRI-defined nutritional risk remained associated with higher odds of prolonged postoperative LOS (OR = 6.31, 95% CI 3.42 to 11.65, *P* < .001). The risk burden score also remained positively associated with prolonged postoperative LOS (OR = 2.76, 95% CI 2.05 to 3.73, *P* for trend <.001).

**Table 5:** Sensitivity analyses.

Sensitivity analysis	SII-GNRI joint exposure group	N	Prolonged postoperative LOS, n (%)	OR (95% CI)	*P* value
SII cutoff changed to median	Non-high SII+no nutritional risk	193	17 (8.8%)	Ref	–
	High SII+no nutritional risk	94	13 (13.8%)	1.51 (0.69, 3.32)	.306
	Non-high SII+GNRI-defined nutritional risk	135	24 (17.8%)	1.90 (0.96, 3.78)	.067
	High SII+GNRI-defined nutritional risk	231	107 (46.3%)	6.31 (3.42, 11.65)	<.001
	SII-GNRI risk burden score, per 1-point increase	–	–	2.76 (2.05, 3.73)	<.001
Postoperative LOS >15 days	Non-high SII+no nutritional risk	249	3 (1.2%)	Ref	–
	High SII+no nutritional risk	38	1 (2.6%)	1.94 (0.19, 19.99)	.579
	Non-high SII+GNRI-defined nutritional risk	241	11 (4.6%)	2.43 (0.64, 9.24)	.191
	High SII+GNRI-defined nutritional risk	125	34 (27.2%)	16.25 (4.57, 57.84)	<.001
	SII-GNRI risk burden score, per 1-point increase	–	–	5.21 (2.93, 9.26)	<.001

Models were adjusted for age, sex, CCI, fracture type, and preoperative waiting time.

CCI = Charlson Comorbidity Index, CI = confidence interval, GNRI = Geriatric Nutritional Risk Index, LOS = length of stay, N/n = number of patients, OR = odds ratio, Ref = reference group, SII = Systemic Immune-Inflammation Index.

When prolonged postoperative LOS was redefined using the 90th percentile cutoff of postoperative LOS (>15 days), high SII plus GNRI-defined nutritional risk remained associated with higher odds of prolonged postoperative LOS (OR = 16.25, 95% CI 4.57–57.84, *P* < .001). The risk burden score showed a persistent graded association (OR = 5.21, 95% CI 2.93–9.26, *P* for trend <.001).

## 4. Discussion

This study showed that higher preoperative SII and lower GNRI were associated with higher odds of prolonged postoperative length of stay in older patients with hip fracture. Patients with both high SII and GNRI-defined nutritional risk had the highest odds, and exploratory OR-based additive interaction estimates were positive. Adding SII-GNRI joint exposure showed improved in-sample discrimination beyond the clinical model, and the findings were generally stable across subgroup and sensitivity analyses. These findings suggest that combined assessment of inflammatory immune burden and nutritional risk may provide additional information for identifying patients with prolonged postoperative LOS.

The association between higher SII and prolonged postoperative LOS is clinically plausible. Older patients with hip fracture often present with trauma-related stress, chronic low-grade inflammation, immune senescence, and coagulation activation. A single inflammatory marker may not fully capture this complex state. SII integrates PLT, NEUT, and LYM, and therefore reflects thrombo-inflammatory activity, innate immune activation, and impaired immune regulation. Long et al showed that admission inflammatory markers, including the C-reactive protein-to-albumin ratio and neutrophil-to-lymphocyte ratio, were associated with long-term mortality in older patients undergoing hip fracture surgery.^[14]^ Liu et al reported that higher SII was associated with increased 30-day and 1-year mortality among critically ill older patients with hip fracture in the Medical Information Mart for Intensive Care database.^[15]^ Çelen found that postoperative day 5 SII independently predicted 1-year mortality after geriatric hip fracture.^[16]^ Chen et al reported in a meta-analysis that elevated preoperative and postoperative neutrophil-to-lymphocyte ratios were associated with long-term mortality after hip fracture surgery.^[17]^ Wang et al also found that a higher platelet-to-lymphocyte ratio predicted poor 1-year survival in older patients with hip fracture.^[18]^ These studies mainly focused on mortality. Our study extends this evidence by showing that inflammatory immune burden may also be reflected earlier as prolonged postoperative LOS.

In this study, high SII was defined using the cohort-specific 75th percentile because no universally accepted clinical threshold for elevated SII has been established in older patients with hip fracture. This percentile-based approach allowed identification of patients with relatively higher inflammatory burden within the study cohort while maintaining an adequate sample size for comparative analyses. However, the selected threshold should be interpreted as a cohort-specific marker of elevated inflammatory burden rather than a universal clinical cutoff.

Lower GNRI was also associated with prolonged postoperative LOS. GNRI incorporates Alb and body weight-related information, and it reflects not only insufficient nutritional intake but also inflammatory burden, metabolic reserve, and tissue repair capacity. Soeters et al emphasized that hypoalbuminemia is closely linked to inflammation, increased vascular permeability, and enhanced catabolism, and should not be interpreted simply as isolated malnutrition.^[19]^ Norman et al highlighted that malnutrition in older adults increases vulnerability through impaired immune function, reduced muscle mass, and limited metabolic adaptation.^[20]^ In the setting of hip fracture, nutritional risk is strongly intertwined with sarcopenia, frailty, and impaired rehabilitation capacity. Yoo et al showed that malnutrition and chronic inflammation were risk factors for sarcopenia in older patients with hip fracture.^[21]^ Inoue et al summarized that undernutrition, sarcopenia, and frailty frequently overlap in fragility hip fracture and jointly affect clinical outcomes.^[22]^ Dagnelie et al further demonstrated that baseline nutritional status independently predicted clinical outcomes and 5-year mortality after hip fracture.^[23]^ Han et al found that inadequate nutrition was common among patients admitted with hip fracture and was associated with prolonged hospital stay, impaired early mobilization, pressure ulcers, in-hospital mortality, and worse discharge destination.^[24]^ In the present study, each 10-point decrease in GNRI was associated with higher odds of prolonged postoperative LOS, suggesting that nutritional risk affects not only long-term survival and complications but also the early pace of recovery.

A key finding of this study was the potential additive interaction between high SII and GNRI-defined nutritional risk. Multiplicative interaction was not statistically significant, and additive interaction measures derived from logistic regression ORs showed positive estimates. However, these findings should be interpreted cautiously because prolonged postoperative LOS was not a rare outcome and OR-based additive interaction measures may not closely approximate risk-based additive interaction measures. Accordingly, the OR-based additive interaction analysis should be regarded as exploratory and hypothesis-generating. Knol and VanderWeele recommended presenting interaction on both multiplicative and additive scales, because additive interaction is particularly relevant for estimating the additional burden attributable to joint exposure.^[25]^ Rothman et al also emphasized that excess risk from coexisting risk factors may be more meaningful for etiologic and public health interpretation than relative effects alone.^[26]^ In our study, high SII alone did not reach statistical significance, GNRI-defined nutritional risk alone was associated with prolonged postoperative LOS, and the coexistence of both factors was associated with markedly higher odds. This pattern may indicate a potential interplay between inflammatory and nutritional pathways during postoperative recovery, although further validation using appropriate risk-based approaches is required. Frenkel Rutenberg et al evaluated several nutritional and inflammatory indices for 90-day mortality after fragility hip fracture surgery and found that the platelet-to-lymphocyte ratio was the only index that differed significantly between mortality groups, highlighting the need to interpret individual indices rather than assume equivalent prognostic value.^[27]^ Mechanistically, inflammation may promote protein breakdown, muscle wasting, and impaired tissue repair, while nutritional risk may reduce immune regulation, wound healing capacity, and tolerance to rehabilitation. SII-GNRI joint exposure may therefore capture a high-risk phenotype characterized by immune activation, nutritional depletion, reduced muscle reserve, and frailty tendency.

The clinical value of SII-GNRI joint assessment lies in its feasibility. Both indices can be obtained from routine preoperative blood tests and basic anthropometric data. In this study, adding SII-GNRI joint exposure showed improved in-sample discrimination beyond the clinical model, suggesting that inflammatory and nutritional information may provide additional risk stratification information. However, this finding should be considered preliminary and hypothesis-generating because internal resampling validation, calibration assessment, decision curve analysis, and external validation were not performed. Schweller et al reported that LOS after hip fracture is affected by patient-related, hospital-related, and surgical factors.^[28]^ Nordström et al showed in a Swedish nationwide cohort that hospital LOS after hip fracture was associated with short-term mortality after discharge, underscoring the clinical importance of the recovery process itself.^[29]^ Grigoryan et al reported in a meta-analysis that orthogeriatric care improved mortality outcomes in patients with hip fracture.^[30]^ Malafarina et al showed that nutritional supplementation improved muscle mass and nutritional indicators in older patients with hip fracture.^[31]^ Takahashi et al reported that nutritional therapy combined with rehabilitation may improve outcomes in older patients with hip fracture.^[32]^ Bui et al further showed that several preoperative factors, including age, ASA classification, sex, residence status, kidney function, dementia, diabetes, and low Hb, were associated with early mortality after hip fracture surgery.^[33]^ These findings support a multidimensional approach to perioperative risk stratification. For patients with both high SII and GNRI-defined nutritional risk, these findings may help identify individuals who could benefit from closer perioperative attention. Potential strategies, including nutritional assessment, inflammation and infection surveillance, anemia and renal function management, and proactive rehabilitation and discharge planning, may represent possible clinical considerations; however, their effectiveness requires confirmation in prospective interventional studies.

Several limitations should be acknowledged. First, this was a single-center retrospective cohort study, and selection bias and residual confounding could not be fully excluded despite adjustment for prespecified clinical covariates. Because inclusion in the analytic cohort required complete key preoperative laboratory and outcome data, the exclusion of patients with incomplete records may have introduced additional selection bias if data completeness was related to underlying clinical status or postoperative course. Second, SII and GNRI were calculated from single preoperative measurements and did not capture dynamic perioperative changes in inflammatory and nutritional status. Third, both the SII cutoff and the postoperative LOS threshold were derived from cohort-specific percentiles. The high SII cutoff (>1102.5) was data-derived and may not be directly generalizable to other populations. Although percentile-based categorization is useful when established clinical thresholds are unavailable, external validation in independent cohorts is required. Prolonged postoperative LOS was defined as postoperative LOS above the cohort-specific 75th percentile. This definition is practical but may be influenced by discharge policy, rehabilitation resources, family support, and institutional care pathways. Fourth, muscle mass, grip strength, gait speed, frailty score, pain control, severity of postoperative complications, and rehabilitation adherence were not available. This limited further clarification of the pathways linking inflammatory-nutritional imbalance to prolonged postoperative LOS. Fifth, RERI, AP, and SI were derived from logistic regression ORs. Because prolonged postoperative LOS was not a rare outcome, these OR-based additive interaction measures may not closely approximate additive interaction measures on the risk scale. Therefore, the observed additive interaction findings should be interpreted cautiously. In addition, the incremental discrimination analysis was performed within the observed cohort, and no internal resampling validation, calibration assessment, decision curve analysis, or external validation was conducted. Therefore, the improvement in AUC should be interpreted as preliminary, hypothesis-generating evidence of improved in-sample discrimination rather than established predictive or clinical utility. Finally, although the analyses provided evidence of a potential additive interaction based on these statistical measures, the observational design cannot establish a biological causal mechanism.

## 5. Conclusions

The combined assessment of SII and GNRI may provide additional information for preoperative risk stratification and may help identify patients who require closer perioperative attention. However, given the single-center retrospective design, cohort-derived SII and postoperative LOS thresholds, and lack of internal and external validation, these findings should be considered preliminary and should not be used as stand-alone criteria for clinical decision-making. Further validation in independent and diverse populations is required. Whether clinical strategies guided by these markers can improve patient outcomes requires prospective evaluation.

## Acknowledgments

The authors thank the clinical staff involved in data collection, data verification, and patient care.

## Author contributions

**Conceptualization:** Jinguang Zhang.

**Data curation:** Jinguang Zhang.

**Formal analysis:** Jinguang Zhang.

**Methodology:** Xiao Yu, Dongxu Sun.

**Supervision:** Xiao Yu.

**Validation:** Xiao Yu.

**Visualization:** Xiao Yu.

**Funding acquisition:** Dongxu Sun.

**Project administration:** Dongxu Sun.

**Writing – original draft:** Jinguang Zhang.

**Writing – review & editing:** Xiao Yu, Dongxu Sun.
